# Upper Extremity Motor Impairments and Microstructural Changes in Bulbospinal Pathways in Chronic Hemiparetic Stroke

**DOI:** 10.3389/fneur.2017.00257

**Published:** 2017-06-13

**Authors:** Meriel Owen, Carson Ingo, Julius P. A. Dewald

**Affiliations:** ^1^Department of Physical Therapy and Human Movement Sciences, Feinberg School of Medicine, Northwestern University, Chicago, IL, United States; ^2^Interdepartmental Neuroscience Program, Northwestern University, Chicago, IL, United States; ^3^Department of Biomedical Engineering, McCormick School of Engineering, Northwestern University, Evanston, IL, United States

**Keywords:** chronic stroke, motor impairment, brainstem, white matter, diffusion imaging

## Abstract

Following hemiparetic stroke, precise, individuated control of single joints is often replaced by highly stereotyped patterns of multi-joint movement, or abnormal limb synergies, which can negatively impact functional use of the paretic arm. One hypothesis for the expression of these synergies is an increased dependence on bulbospinal pathways such as the rubrospinal (RubST) tract and especially the reticulospinal (RetST) tracts, which co-activate multiple muscles of the shoulder, elbow, wrist, and fingers. Despite indirect evidence supporting this hypothesis in humans poststroke, it still remains unclear whether it is correct. Therefore, we used high-resolution diffusion tensor imaging (DTI) to quantify white matter microstructure in relation to severity of arm synergy and hand-related motor impairments. DTI was performed on 19 moderately to severely impaired chronic stroke individuals and 15 healthy, age-matched controls. In stroke individuals, compared to controls, there was significantly decreased fractional anisotropy (FA) and significantly increased axial and radial diffusivity in bilateral corona radiata and body of the corpus callosum. Furthermore, poststroke, the contralesional (CL) RetST FA correlated significantly with both upper extremity (UE) synergy severity (*r* = −0.606, *p* = 0.003) and hand impairment (*r* = −0.609, *p* = 0.003). FA in the ipsilesional RubST significantly correlated with hand impairment severity (*r* = −0.590, *p* = 0.004). For the first time, we separately evaluate RetST and RubST microstructure in chronic stroke individuals with UE motor impairment. We demonstrate that individuals with the greatest UE synergy severity and hand impairments poststroke have the highest FA in the CL RetST a pattern consistent with increased myelination and suggestive of neuroplastic reorganization. Since the RetST pathway microstructure, in particular, is sensitive to abnormal joint coupling and hand-related motor impairment in chronic stroke, it could help test the effects of specific, and novel, anti-synergy neurorehabilitation interventions for recovery from hemiparesis.

## Introduction

Approximately 85% of stroke survivors experience significant motor impairment in the contralesional (CL) arm (1), which can include a loss of independent joint control (2, 3), weakness (4), and spasticity (5). After stroke, precise, individuated control of single joints is often replaced by highly stereotyped patterns of multi-joint movement caused by abnormal muscle co-activation patterns (6). The most prevalent of these patterns is the flexion synergy, which is characterized by an abnormal coupling of shoulder abduction and elbow, wrist, and finger flexion (7, 8). This impairment has a negative impact on reaching ability (9) and hand function (3, 10), both critical components of functional use of the arm during activities of daily living. Despite the debilitating nature of this motor impairment, the underlying neuropathophysiology is not fully understood.

One hypothesis for why the flexion synergy emerges is that following a reduction of corticofugal input from the lesioned hemisphere, there is an increased dependence on CL motor cortex and bulbospinal pathways, such as reticulospinal (RetST) and rubrospinal (RubST) tracts. Therefore, in the present study, we quantify microstructural properties in white matter of both the brain and the brainstem, focusing primarily on corticoreticulospinal and corticorubrospinal systems. We evaluate whether these microstructural properties increase in integrity in relation to arm synergy and hand impairment severity, which could be indicative of increased use.

Although the RetST was previously believed to be predominantly involved in gross movements, such as locomotion (11, 12) and posture (13, 14), recent work in primates suggests the RetST also influences the motor neurons that control forearm and intrinsic hand muscles (15). In the non-human primate, stimulation of the RetST produces ipsilateral wrist flexor, elbow flexor, and shoulder abductor activation (16), mirroring the flexion synergy pattern observed in humans poststroke. Furthermore, stimulating the RetST after a corticospinal tract (CST) lesion elicits increased excitatory post-synaptic potentials in motoneurons innervating the forearm flexor and intrinsic hand muscles (17). This evidence makes the contralesional corticoreticulospinal system a compelling candidate for underlying abnormal joint coupling in humans with hemiparetic stroke.

In the non-human primate, the RubST also contributes to reaching and grasping movements (18) and has been shown to be important in recovery of hand function after CST damage (19, 20). One study showed that increased white matter integrity in bilateral red nucleus (RN) correlated with worse clinical outcomes in humans with chronic stroke (21); however, the RubST has been reported as relatively insignificant in humans (22, 23). The evidence for whether the RetST and the RubST contribute to abnormal joint coupling and hand impairment in humans poststroke still remains indirect and inconclusive.

We used high-resolution diffusion tensor imaging (DTI) (24) tract-based spatial statistics (TBSS) (25) to perform a voxel-wise comparison of white matter microstructure between stroke and control individuals. We analyzed fractional anisotropy (FA), a measurement typically associated with tract integrity, as well as axial diffusivity (AD) and radial diffusivity (RD), which represent diffusion parallel and perpendicular to the principle direction of diffusion, respectively. Because previous studies have reported altered diffusion properties in lesioned tissue (26–28), we excluded potential lesion-compromised voxels from our TBSS analysis to assess changes in normal-appearing white matter. We used the TBSS-derived white matter skeleton to investigate whether microstructural tissue properties within specific regions of the brainstem (CST, RetST, RubST) and subcortical white matter within CL motor areas [primary motor area (M1), premotor area (PM), supplementary motor area (SMA), body of the corpus callosum] are sensitive to upper extremity (UE) motor impairment in chronic stroke individuals.

We evaluated UE motor impairment using the Fugl-Meyer Assessment (FMA), a stroke-specific, performance-based motor impairment index, which measures impairments, such as loss of independent joint function, stretch reflex hyper-excitability, and altered sensation (29). It is one of the most widely used clinical scales of motor impairment poststroke (30). While previous studies have looked at diffusion MRI metrics in relation to the entire FMA score (31, 32), we used only the UE measurements of arm synergies and hand function to determine whether microstructural properties in specific white matter regions of interest (ROIs) were correlated.

In the present study, we hypothesized that microstructural integrity in specific regions of the extrapyramidal brainstem would be increased in chronic stroke in a manner sensitive to synergy and hand-related impairment severity. We demonstrate a significant decrease in FA in bilateral corona radiata and body of the corpus callosum in chronic stroke when compared to controls; however, within stroke subjects, specific brainstem regions show the highest FA in individuals with the most synergy-driven arm and hand impairment. More precisely, we describe the relation between CL RetST integrity and both expression of synergy and hand impairment and between ipsilesional (IL) RubST integrity and hand impairment in chronic hemiparetic stroke individuals.

## Materials and Methods

### Patients and Healthy Controls

Nineteen moderately to severely impaired stroke individuals (15 M, 4 F; average age 59 years, SD 8 years; 9 severe, 10 moderate) and 15 age-matched healthy controls (8 M, 7 F; average age 61 years, SD 7 years) without known neurological abnormalities were included in the study. Stroke subjects sustained a unilateral brain lesion at least 4 months prior to participation in the study. Stroke participants were selected from the Clinical Neuroscience Research Registry, housed in the Rehabilitation Institute of Chicago, and from individuals residing in Chicago who wished to participate.

Inclusion criteria for stroke individuals were as follows: (1) paresis confined to one side, with motor impairment of the upper limb, (2) an overall UE FMA score between 0 and 50 out of 66 [0–20 = severe, 21–50 = moderate (33)], (3) absence of severe cognitive or affective dysfunction, and (4) absence of severe concurrent medical problems. The protocol was approved by the Northwestern University Institutional Review Board, and all subjects provided a written, informed consent in accordance with the Declaration of Helsinki. Table 1 shows the clinical characteristics of the stroke individuals.

**Table 1:** Patient demographics and clinical characteristics of all stroke subjects enrolled in the study.

	Age (years)	Time post stroke (months)	Lesioned hemi	Lesion location	UE-FMA total	FMA synergy	FMA hand
S01	61–65	237	R	IC, BG, Thal	11	9	2
S02	46–50	209	L	IC, BG, Thal	17	9	2
S03	61–65	82	R	IC, BG	14	10	0
S04	66–70	26	R	Par, Occ, IC	15	10	1
S05	71–75	160	R	IC	15	9	2
S06	61–65	359	L	IC, BG, Thal	16	10	2
S07	51–55	5	L	Par, IC	21	11	2
S08	66–70	246	L	IC, BG, Thal	17	12	1
S09	61–65	83	R	IC, Pons	17	11	3
S10	61–65	96	R	IC, BG	19	10	1
S11	56–60	100	R	Par, IC, BG, Thal	22	17	1
S12	56–60	138	R	Occ, IC	22	18	3
S13	61–65	90	L	IC	24	14	2
S14	66–70	95	L	Thal	29	14	6
S15	56–60	52	L	IC, BG	29	18	3
S16	35–40	106	R	Par, IC, Thal	29	17	5
S17	51–55	95	R	Occ, IC, Thal	36	22	6
S18	61–65	106	L	IC, Thal	32	19	7
S19	46–50	59	R	IC, BG, Thal	33	18	8

*BG, basal ganglia; FMA, Fugl-Meyer assessment; IC, internal capsule; Occ, occipital lobe; Par, parietal lobe; Thal, thalamus; UE, upper extremity*.

### Clinical Assessment

Upper extremity FMA was performed by a licensed physical therapist to evaluate motor impairment in stroke individuals. Higher values indicated less impairment (29). From this assessment, arm synergies (range: 9–22 out of a maximum score of 30) and hand-related impairments (range: 0–8 out of a maximum score of 24) were calculated separately. In our subsequent analysis, we determined which neural regions were correlated to synergy-related arm impairment or hand impairment.

### Data Acquisition

MRI scans were performed at Northwestern University’s Center for Translation Imaging on a 3-T Siemens Prisma scanner with a 64-channel head coil. Structural T1-weighted scans were acquired using an MPRAGE sequence [TR = 2.3 s, TE = 2.94 ms, field of view (FOV) = 256 mm × 256 mm] producing an isotropic voxel resolution of 1 mm × 1 mm × 1 mm and lasting 10 min. Diffusion-weighted images were collected from all subjects using spin-echo echo-planar imaging (TR = 5 s, TW = 85 ms, matrix size = 150 × 150, FOV = 225 mm × 225 mm, slice thickness = 1.5 mm, interslice gap = 0 mm, number of slices = 120) producing an isotropic voxel resolution of 1.5 mm × 1.5 mm × 1.5 mm and lasting 5 min. The sequence consisted of diffusion weighting of 1,000 s/mm^2^ in 60 different directions and 8 scans with no diffusion weighting (*b* = 0 s/mm^2^). Visual inspection of acquired images was performed immediately following the data acquisition to guarantee no artifacts and stable head position.

### DTI Preprocessing

The diffusion-weighted images were first brain-extracted using the brain extraction toolbox in FMRIB software Library (FSL) (http://www.fmrib.ox.ac.uk/fsl). The data were then denoised using an estimate of the noise variance in CSF signal intensity of the right ventricle (34) and Rician noise corrected (35). The data were corrected for motion and eddy currents by co-registering diffusion-weighted images to the image acquired with *b* = 0 s/mm^2^ using the FLIRT toolbox in FSL. The motion correction transformation matrix was applied to the diffusion gradient directions to rotate them according to the registration algorithm. The preprocessed diffusion-weighted data were fitted to a tensor on a voxel-wise basis using DTIFIT in the FSL Diffusion Toolbox (36).

### TBSS Analysis

For those individuals with lesions in the left hemisphere, FA maps were flipped so that all subjects had lesions in the right hemisphere and group analysis compared all CL hemispheres in the left hemisphere. FA maps were first linearly and then non-linearly registered to the FMRIB58_FA in Montreal Neurological Institute’s (MNI) standard space. A mean FA image was then created from all individual FA images and used to generate a common group skeleton. A threshold was applied at 0.2 to minimize potential white matter/gray matter partial volume effects. Finally, each FA image was projected onto the common group skeleton for subsequent statistical analysis. The same transformations were applied to both AD and RD maps, which represent diffusion parallel and perpendicular to the principal direction of diffusion, respectively.

### Lesion Mask

Lesion masks were generated on the T1-weighted scans using automated pipelines developed at Northwestern University (37). For those individuals with lesions in the left hemisphere, lesion masks were flipped into the right hemisphere so that group analysis compared all CL hemispheres in the left hemisphere. For each subject, T1 images were brain extracted and affine registered to the *b* = 0 s/mm^2^ diffusion scan using FSL FLIRT (38, 39). The same transformation matrix was applied to align individual lesion masks to the subject-specific diffusion image, with the assumption that hypointensity due to the lesion in the T1-weighted image matched the hyperintensity due to the lesion in the T2-weighted *b* = 0 s/mm^2^ image. The lesion mask for each subject was transformed into MNI space, utilizing the same transform applied to the FA images. A cumulative right hemisphere lesion mask was created for all stroke individuals by adding individual lesion masks from each subject in standard space. This common lesion mask was used to exclude all lesioned voxels from subsequent TBSS analysis and to ensure statistical testing was performed only on normal appearing white matter, with regard to T1-weighted signal, in all subjects.

### TBSS Statistical Analysis

In order to determine voxel-wise statistics between stroke subjects and healthy controls, permutation testing was applied to the 4D skeletonized FA, AD, and RD maps, with lesioned voxels excluded. Using RANDOMISE in FSL, 5,000 permutations with threshold-free cluster enhancement were performed to correct for multiple comparisons (40).

### ROI Analysis

**Figure 1:**
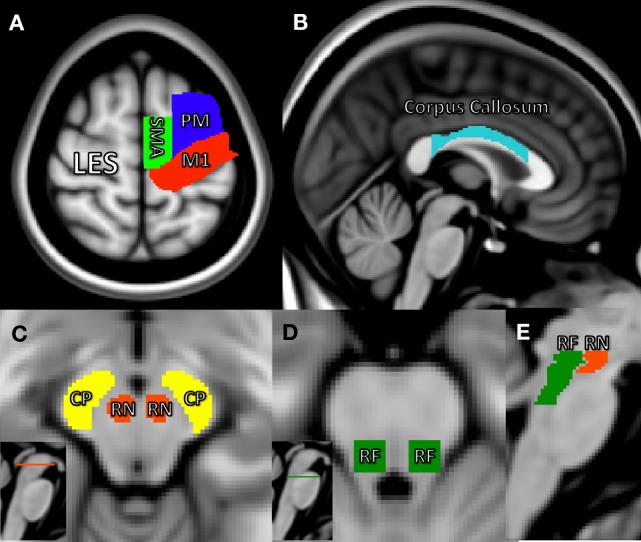
Region of interest masks in Montreal Neurological Institute’s space. **(A)** Primary motor area (red), supplementary motor area (green), premotor area (blue), **(B)** body of the corpus callosum (light blue), **(C)** horizontal midbrain cross-section showing cerebral peduncle (CP) portion of the corticospinal tract (yellow) and red nucleus (RN) (red), **(D)** horizontal pontine cross-section showing reticular formation (RF) (green), and **(E)** sagittal brainstem showing RF including reticulospinal (green) and RN including rubrospinal tracts (red).

A ROI analysis was performed using the TBSS-generated white matter skeleton to further quantify diffusion characteristics in a specific subset of neural regions relevant to motor recovery poststroke. These included CL white matter from motor regions: primary motor area (M1), premotor area (PM), supplementary motor area (SMA), and body of the corpus callosum as shown in Figures 1A,B and bilateral brainstem regions, which included cerebral peduncles (CPs), containing descending projections of the CST, reticular formation (RF) containing part of the descending RetST, and red nucleus (RN) containing descending RubST projections, shown in Figures 1C–E. The CP and corpus callosum masks were obtained from the JHU ICBM-DTI-81 White-Matter Atlas (41–43); the motor region masks were obtained from Human Motor Area Template (44); and the RN and RF masks were drawn manually in the midbrain and pons based on known anatomy (45) and extended caudally to capture descending projections. The midbrain region was carefully selected because at this level, CP, RF, and RN are visibly separable (46). Average FA values were calculated from all white matter skeleton voxels within each ROI: left CP, right CP, left RetST, right RetST, left RubST, right RubST, white matter from motor regions (M1, PM, SMA), and body of the corpus callosum.

### ROI Statistical Analysis

If an ROI did not show significant differences in TBSS, the stroke group was split into severe and moderate based on FMA score [0–20 = severe, 21–50 = moderate (33)], for further analysis in which a one-way ANOVA and *post hoc* testing were performed on the control, moderate stroke, and severe stroke group ROI values. Furthermore, a Spearman correlation analysis was carried out between both hand and arm components of the UE-FMA and average FA values within each ROI: left and right CP, left and right RetST, and left and right RubST, white matter from CL motor regions (M1, PM, SMA) and body of the corpus callosum across stroke participants. If there was no significant correlation for FA and impairment within an ROI, AD and RD were analyzed for possible correlation to impairment. For all non-significant correlations between FA and impairment presented in Table 2, correlations for AD and RD were also non-significant. We accounted for multiple comparisons using a Bonferroni correction. An overall alpha-level of 0.005 was considered significant, which was obtained by dividing alpha of 0.05 by the 10 comparisons that were made. There was a significant correlation between synergy and hand FMA components (*r* = 0.642, *p* = 0.0015).

**Table 2:** *r* and *p* values for correlations between upper extremity FMA (synergy and hand) and fractional anisotropy values in specific brain regions.

	Synergy		Hand	
	*r*	*p*	*r*	*p*
Ipsilesional CP	−0.089	0.357	−0.132	0.295
Contralesional CP	−0.531	***0.010***	−0.520	***0.011***
Ipsilesional RetST	−0.159	0.257	−0.259	0.142
Contralesional RetST	−0.606	**0.003**	−0.609	**0.003**
Ipsilesional RubST	−0.282	0.121	−0.590	**0.004**
Contralesional RubST	−0.349	0.072	−0.386	0.051
Contralesional M1	−0.102	0.339	−0.140	0.284
Contralesional PM	−0.257	0.144	−0.015	0.475
Contralesional SMA	−0.415	***0.039***	−0.142	0.281
Corpus callosum	−0.502	***0.014***	−0.364	0.063

*CP, cerebral peduncle; RetST, reticulospinal tract; RubST, rubrospinal tract; M1, primary motor tract; PM, premotor tract; SMA, supplementary motor tract*.

*Bold signifies reaching statistical significance of p < 0.005*.

*Bold-italic signifies trends such that 0.005 < p < 0.05*.

## Results

### Lesion Mask

**Figure 2:**
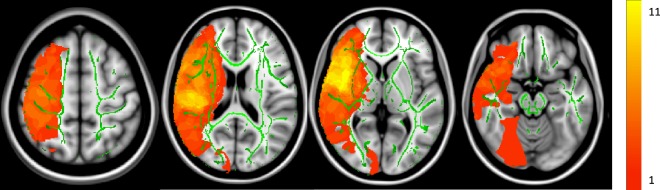
Cumulative lesion mask for all stroke subjects. *Z* = 126, 90, 75, and 55. The color bar indicates how many subjects sustained a lesion in that particular voxel. The maximum was 11 subjects sharing a single-lesioned voxel with a minimum of one individual. The average group white matter skeleton is shown in green.

Figure 2 shows the cumulative lesion mask for all stroke individuals. Areas that appear in yellow signify a higher number of subjects had a lesion in that voxel. Voxels in which at least one individual had a lesion were excluded for subsequent TBSS analysis.

### TBSS Analysis Stroke vs. Control

**Figure 3:**
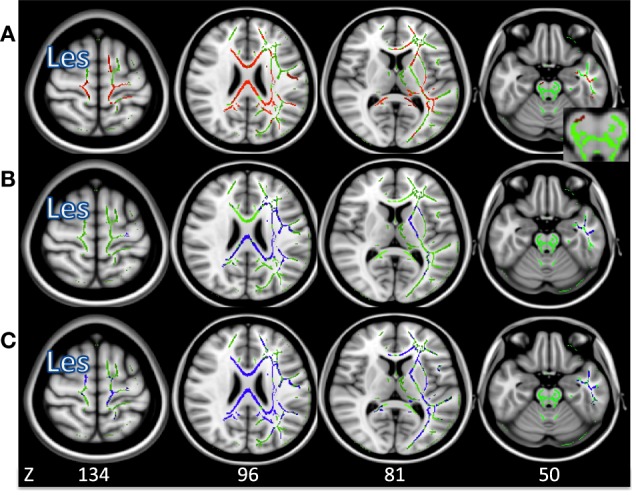
**(A)** Voxels in which fractional anisotropy is decreased in stroke compared to control (*p* < 0.05) shown in red. **(B)** Voxels in which axial diffusivity is increased in stroke compared to control (*p* < 0.05) shown in blue. **(C)** Voxels in which radial diffusivity is increased in stroke compared to control (*p* < 0.05) shown in blue. The average group skeleton is shown in green. Les signifies the lesioned hemisphere.

Figures 3A–C shows the average group white matter skeleton in green, with lesion-compromised voxels removed in the right hemisphere. In Figure 3A, there is significantly decreased FA in regions of the corona radiata, corpus callosum, and IL CP in stroke when compared to controls, shown in red. Figures 3B,C show there was significantly increased AD and RD in motor regions of the corona radiata, internal capsule, and corpus callosum in stroke when compared to controls, shown in blue.

**Figure 4:**
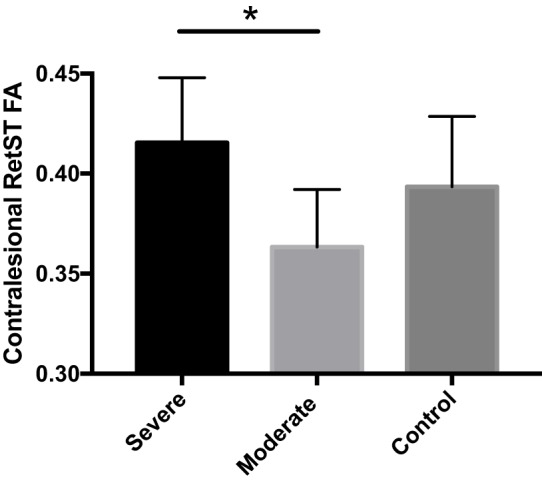
Average contralesional reticulospinal tract (RetST) fractional anisotropy (FA) for severe stroke group (0.415), moderate stroke group (0.363), and control group (0.393). Severely impaired individuals show significantly greater FA (*p* = 0.0045) than moderately impaired individuals. SD bars are shown. 95% confidence intervals were severe [0.39, 0.43], moderate [0.34, 0.38], and control [0.37, 0.41].

In contrast, the CL CP, bilateral RetST, and bilateral RubST ROIs showed no significant group differences in the TBSS analysis and were further analyzed by subdividing the stroke group. When dividing the stroke group into moderately (FMA 21–50) and severely impaired (FMA 0–20) groups, there was no significant effect of group on the diffusion measures of the CL CP or bilateral RubST. However, there was a statistically significant difference between groups as determined by one-way ANOVA for FA in CL RetST (*p* = 0.0063). *Post hoc* testing revealed that FA in CL RetST was significantly higher in severely impaired individuals (0.41 ± 0.03, *p* = 0.0045) compared to moderately impaired individuals (0.36 ± 0.02). This result was primarily driven by differences in RD (*p* = 0.024) rather than AD (*p* = 0.866). The average FA value for the control group (0.39 ± 0.03) was between the average FA values of the moderately and severely impaired individuals, but statistical significance was not reached for these comparisons [severe vs. control (*p* = 0.243), moderate vs. control (*p* = 0.093)]. These results are shown in Figure 4.

### Correlation Analysis within Stroke

**Figure 5:**
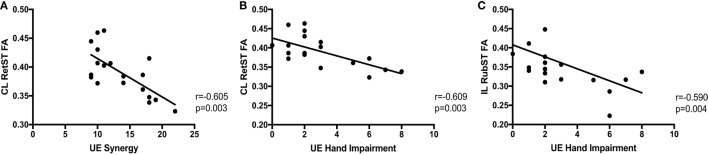
Correlations between impairment and brainstem white matter microstructure. Upper extremity (UE) synergy expression is correlated with **(A)** fractional anisotropy (FA) in contralesional (CL) reticulospinal tract (RetST) (95% CI [−0.83, −0.19]). UE hand impairment is correlated with **(B)** FA in CL RetST (95% CI [−0.83, −0.20]) and **(C)** FA in ipsilesional (IL) rubrospinal tract (RubST) (95% CI [−0.82, −0.17]).

A correlation analysis was performed for the synergy and hand components of the UE FMA and the ROIs thought to be involved in motor recovery, shown in Table 2. There were significant negative correlations between synergy expression in the arm and CL RetST (Figure 5A), indicating that individuals with the most synergy expression had the highest FA in this region. There was a trend of a negative correlation between synergy expression and FA of the CL CP, CL SMA white matter and body of the corpus callosum, and no significant correlations between synergy expression and FA of IL CP, IL RetST, bilateral RubST, CL M1, or CL PM areas (Table 2). There were no significant correlations between FA in IL M1, PM, and SMA white matter and impairment severity. Additionally, since there was a significant correlation between synergy and hand FMA components, we cannot consider the results independent, but related as the significance patterns of correlations across brain regions demonstrate in Table 2.

There was a significant correlation between hand impairment scores and FA in CL RetST (Figure 5B) and IL RubST (Figure 5C). There was a trend of negative correlation between hand impairment and FA of the CL CP. No significant correlations were found between hand scores and FA in bilateral CP, IL RetST, CL RubST, CL M1, PM, SMA white matter, or corpus callosum.

## Discussion

### Summary of Results

Our results support previous reports of decreased FA and increased AD and RD in white matter of motor regions, corpus callosum, and IL CP poststroke compared to healthy controls (32, 47), a pattern reflective of chronic white matter degeneration. We extend these findings by showing that in chronic hemiparetic stroke, microstructure in the CL RetST was related to both synergy and hand impairment, and microstructure in the IL RubST was related to hand impairment. Individuals with the most severe synergy-driven impairment had higher FA in CL RetST when compared to moderately impaired individuals, a pattern which reflects plastic remodeling in healthy controls (48). Our findings also show that the IL RubST may play a role in the expression of hand impairment in more impaired individuals with chronic stroke and emphasize the importance of including brainstem morphology into models of neural reorganization poststroke.

### Specific Changes in Brainstem White Matter in Chronic Hemiparetic Stroke

#### Contralesional Reticulospinal Tract

Previous studies have used diffusion MRI to show that decreased FA in lesioned CST and corpus callosum are sensitive to impairment poststroke (32, 49). In the present study, we included the brainstem and identified a region in the CL RetST, which showed increased FA in the most severely impaired individuals (Figure 4), a difference primarily driven by a decrease in RD, shown to be related to myelination in animal models (50).

Impairment-dependent differences within stroke have been described in previous studies. For example, in moderately impaired individuals, shoulder abduction reduced reaching distance and voluntary hand opening, but in severely impaired individuals performing the same task, while lifting the arm, reaching became almost impossible and grasping forces were generated at the hand (10, 51, 52). This finding could be indicative of damage-dependent reorganization in the most severely impaired individuals, who may need to rely more heavily on CL RetST to elicit movement, albeit synergistic and dysfunctional in nature, poststroke.

Initial work characterizing the RetST focused on its role in gross movements, such as locomotion (53), and postural stability (54); however, recent studies in primates show that the RetST makes mono- and di-synaptic connections to motor neurons controlling muscles of the forearm (15). Interneurons involved in controlling the hand often receive convergent information from both CST and RetST (55), and stimulating the RetST elicits excitatory post-synaptic potentials in hand muscles (56). Historically, it has been assumed that the CST almost exclusively controls the hand, but anatomical evidence in primates suggests a more nuanced model of forearm and hand control in which RetST contributes less selective input, and CST provides much stronger, more fractionated and precise commands (3).

Including the RetST contributions in our understanding of hand control could help inform what happens when there is significant stroke-related damage to the CST. Stimulation of the RF in a non-human primate produces ipsilateral elbow, wrist and finger flexor, and shoulder abductor activation (16), reflecting the role of the RetST in simultaneously influencing multiple motor neurons and mirroring the flexion synergy pattern observed in humans poststroke. Additionally, following a CST lesion in primates, RetST connections to both forearm and hand muscles selectively strengthen (17). If the CST is damaged, the CL corticoreticulospinal pathway may possess the properties required to help drive motor neurons and interneurons controlling the forearm in the spinal cord, but this could come at the cost of independent control of individual joints. This anatomical and physiological evidence supports the hypothesis that the RetST is a potential pathway that could explain the development of the flexion synergy poststroke.

#### Ipsilesional Rubrospinal Tract

In non-human primates, the magnocellular subdivision of the RN gives rise to the RubST, which decussates and descends down to the contralateral spinal cord. Cells in the RN receive input from primary motor cortex (57). In the absence of the CST, animals with a lesioned RubST lose the ability to grasp (19), and output from the RubST has been shown to strengthen after unilateral CST lesion (20). Despite this evidence from non-human primates, it has been argued that humans do not have a significant RubST descending from the midbrain (22); however, a new tractography study has been able to trace this pathway *in vivo* (58). Other recent DTI studies in human stroke individuals show a gradual increase in FA in IL RN during stroke recovery (31), and increased FA in bilateral RN when compared to controls, which is correlated with worse clinical outcomes (21). We build on these findings by demonstrating that microstructural integrity of the IL RubST is significantly related to hand impairment. This is anatomically consistent with animal evidence, which supports a role for RubST in contralateral hand-grip. Following a loss of CST, the RubST may provide an additional means to preserve finger flexion, particularly for individuals with the most severe UE impairments.

#### Additional Regions Sensitive to Motor Impairment Poststroke

We observed a trend of negative correlations between UE synergy expression and FA in CL CP, corpus callosum, and white matter projections from CL SMA. Non-human primate work has demonstrated that, of the three cortical motor areas (M1, PM, SMA), direct stimulation of premotor regions (PM, SMA) can result in ipsilateral activity in proximal muscles of the UE, with significantly more responses following SMA stimulation (59). The latency of these responses suggested that they were more likely to be polysynaptic, and anterograde tracers showed a direct projection from SMA to labeled RetST cells (60). TMS studies support the presence of upregulated polysynaptic pathways ipsilateral to the paretic limb in humans poststroke (61, 62). The CL corticoreticulospinal pathway may serve as an alternative, indirect route to access motoneurons poststroke, particularly in the most severely impaired individuals; however, our results suggest that white matter diffusion properties in SMA are not as sensitive to impairment severity as diffusion properties in the brainstem.

Surprisingly, FA of the IL CP, containing the CST, was not sensitive to either arm synergy or hand-related impairment. However, there has not been an established consensus in previous works regarding the IL CST and impairment severity (26, 28, 49, 63–67). Some studies tested whether FA values along the CST relate to functional recovery during an intervention (26, 28, 47, 64). In contrast, our study focused on moderately to severely impaired chronic individuals and synergy severity in the upper limb. Another study showed that greater FA asymmetry in the internal capsule was associated with poorer upper-limb function, but only for individuals who did not demonstrate motor evoked potentials (MEPs) using transcranial magnetic stimulation. In patients with MEPs, FA asymmetry had no predictive power for clinical score (26), showing the relation to FA asymmetry was only present in a subset of patients studied. Another study found an insignificant trend between FA in the CST and baseline motor deficits, and these values did not predict the response to unilateral arm training (64).

Some studies have shown a relationship between FA of the CST and motor skill in chronic stroke (63, 65). These studies had key differences, such as using a clinical test that measures finger function (63), including lesioned voxels (65), and focusing on individuals with good recovery (68). However, a number of studies support our findings, in that they did not find any significant relationship between FA in the IL CST and motor impairment (49, 66, 67). The exact role of microstructural status of the IL CST in long-term impairment in chronic stroke individuals remains unclear. This further emphasizes the importance of understanding the role of all descending motor pathways in relation to recovery, or lack thereof, after stroke.

#### Interpreting Diffusion Property Changes

Fractional anisotropy is sensitive to neural microstructural architecture, but cannot identify the precise biological sources of diffusion changes. By including other diffusion metrics, such as AD and RD, we can more specifically describe observed microstructural changes *in vivo*. Animal models have suggested that AD may be sensitive to axonal damage, whereas RD has been linked to myelin integrity (50). A recent study showed that FA was significantly correlated with myelin basic protein, suggesting that FA is also highly sensitive to myelination (69).

In stroke when compared to control, we found decreased FA—driven by increases in AD and RD—in bilateral motor regions, body of the corpus callosum, and IL CP, which could be reflective of chronic degenerative changes (70); however, a decrease in FA after training has also been reported (71). Because we removed lesioned voxels from our TBSS analysis, we can be more confident that these results are reflective of changes in non-lesioned tissue, indicating that microstructure is altered even in normal-appearing white matter. Since the majority of our participants sustained their strokes for years, or even decades, prior to the study, our findings demonstrate that these widespread microstructural changes are maintained long-term.

In contrast to the widespread decrease in FA, we identified a region in the extrapyramidal brainstem, which shows increased FA in the most severely impaired individuals. This increase in FA was primarily driven by a decrease in RD, with AD showing no significant between-group difference, suggesting that the change could be myelin driven. This finding may be indicative of neuroplastic reorganization in individuals who rely more heavily on brainstem pathways to elicit movement poststroke.

### Limitations

Following stroke, impairment of motor function is one of the most serious consequences. Thus, we need better tools that can help us predict motor impairment in the paretic limb, such as more quantitative peripheral measurements of impairment. In addition, although we have carefully tried to identify and isolate specific pathways, the brainstem contains complex architecture and tightly packed structures. The crus cerebri of the CP contains corticobulbar pathways and pontine projections, and the dorsal pons includes other pathways, such as the tectospinal tract. Future work will combine more quantitative metrics of impairment with higher resolution brainstem imaging and include stroke groups with and without synergy expression to further elucidate the mechanisms underlying abnormal joint torque coupling poststroke.

### Conclusion

Our findings demonstrate that different neural regions may serve as potential backup systems, depending on the level of motor impairment poststroke. Previous studies, using diffusion MRI, have focused on the role that the CST and the corpus callosum play in recovery, but the microstructural properties of brainstem motor pathways, specifically the corticoreticulospinal and corticorubrospinal systems, have not been studied separately *in vivo* in chronic hemiparetic stroke. Our results highlight the importance of including the brainstem motor pathways in models of neural reorganization. They provide potential new research-relevant biomarkers, which are sensitive to synergy- and hand-related motor impairments in chronic hemiparetic stroke. Higher resolution diffusion imaging and detailed atlases will be instrumental in better defining the basic anatomy and connectivity of the human brainstem and determining how it is affected by neural injury. Our study demonstrates the complex, heterogeneous patterns of morphological neural changes as a function of motor impairment level and emphasizes the need for understanding which systems are spared or reorganized after stroke. The current findings provide a framework for the future exploration of the effect of anti-synergy interventions (72, 73) that may promote the maximal utilization of spared corticospinal resources in the lesioned hemisphere. This may lead to largely avoiding or reversing structural and functional changes to indirect CL motor pathways, thus minimizing the devastating effects of the flexion synergy on functional use of the paretic arm after stroke.

## Ethics Statement

This study was carried out in accordance with the recommendations of Northwestern University IRB with written informed consent from all subjects. All subjects gave written informed consent in accordance with the Declaration of Helsinki. The protocol was approved by the Northwestern University IRB.

## Author Contributions

MO and JD: study design. MO and CI: acquisition, analysis. MO, CI, and JD: data interpretation and manuscript preparation.

## Conflict of Interest Statement

The authors declare that the research was conducted in the absence of any commercial or financial relationships that could be construed as a potential conflict of interest. The reviewer, WO, and handling editor declared their shared affiliation, and the handling editor states that the process nevertheless met the standards of a fair and objective review.

## Abbreviations

AD, axial diffusivity; BET, brain extraction toolbox; CP, cerebral peduncle; CST, corticospinal tract; DTI, diffusion tensor imaging; FA, fractional anisotropy; FMA, Fugl-Meyer assessment; FOV, field of view; FSL, FMRIB software Library; M1, primary motor cortex; MNI, Montreal Neurological Institute; PM, premotor cortex; RD, radial diffusivity; RetST, reticulospinal tract; RF, reticular formation; RN, red nucleus; ROI, region of interest; RubST, rubrospinal tract; SMA, supplementary motor area; TBSS, tract-based spatial statistics.
